# A Novel Optical Tissue Clearing Protocol for Mouse Skeletal Muscle to Visualize Endplates in Their Tissue Context

**DOI:** 10.3389/fncel.2019.00049

**Published:** 2019-02-27

**Authors:** Marion Patrick Ivey Williams, Matteo Rigon, Tatjana Straka, Sarah Janice Hörner, Manfred Thiel, Norbert Gretz, Mathias Hafner, Markus Reischl, Rüdiger Rudolf

**Affiliations:** ^1^Institute of Molecular and Cell Biology, Mannheim University of Applied Sciences, Mannheim, Germany; ^2^Interdisciplinary Center for Neurosciences, Heidelberg University, Heidelberg, Germany; ^3^Department of Anesthesiology and Surgical Intensive Care Medicine, Medical Faculty Mannheim, Heidelberg University, Mannheim, Germany; ^4^Medical Faculty Mannheim, Medical Research Center, Heidelberg University, Mannheim, Germany; ^5^Medical Faculty Mannheim, Institute of Medical Technology, Mannheim University of Applied Sciences, Mannheim, Germany; ^6^Institute for Automation and Applied Informatics, Karlsruhe Institute of Technology, Eggenstein-Leopoldshafen, Germany; ^7^Institute of Toxicology and Genetics, Karlsruhe Institute of Technology, Eggenstein-Leopoldshafen, Germany

**Keywords:** acetylcholine receptor, hydrogel embedding, NMJ, optical tissue clearing, skeletal muscle

## Abstract

Neuromuscular junctions (NMJs) mediate skeletal muscle contractions and play an important role in several neuromuscular disorders when their morphology and function are compromised. However, due to their small size and sparse distribution throughout the comparatively large, inherently opaque muscle tissue the analysis of NMJ morphology has been limited to teased fiber preparations, longitudinal muscle sections, and flat muscles. Consequently, whole mount analyses of NMJ morphology, numbers, their distribution, and assignment to a given muscle fiber have also been impossible to determine in muscle types that are frequently used in experimental paradigms. This impossibility is exacerbated by the lack of optical tissue clearing techniques that are compatible with clear and persistent NMJ stains. Here, we present MYOCLEAR, a novel and highly reproducible muscle tissue clearing protocol. Based on hydrogel-based tissue clearing methods, this protocol permits the labeling and detection of all NMJs in adult hindleg extensor digitorum longus muscles from wildtype and diseased mice. The method is also applicable to adult mouse diaphragm muscles and can be used for different staining agents, including toxins, lectins, antibodies, and nuclear dyes. It will be useful in understanding the distribution, morphological features, and muscle tissue context of NMJs in hindleg muscle whole mounts for biomedical and basic research.

## Introduction

Vertebrate NMJs are the synapses between cholinergic motor neurons and skeletal muscle fibers that mediate voluntary muscle contraction. They are embedded in a complex of many different cellular tissue components, with their pre- and postsynaptic apparatuses juxtaposed onto each other. Depending on the species, NMJs are about 10–50 μm in diameter and come in varying forms, such as grape-, plaque-, or pretzel-shaped structures (Lu and Lichtman, 2007). The latter design is of particular interest as it is prevalent in rodent NMJs and is often used as an indicator for neuromuscular disorders or other disease states when fragmentation, simplification, growth, shrinkage, and other such deviations occur (Lyons and Slater, 1991; Valdez et al., 2010; Carnio et al., 2014; Rudolf et al., 2014). In healthy adult mouse muscle, each syncytial fiber is roughly 50 μm in diameter and up to 4 cm in length; it contains thousands of myonuclei and is precisely innervated by one NMJ (Krause, 1863). This is different during embryonic development and the perinatal stages, where most muscle fibers are contacted by more than one neuron (Lee et al., 2017). This sort of poly-innervation is corrected within the first 2 to 3 postnatal weeks. Nevertheless, it can reappear during denervation—reinnervation cycles and other non-physiological conditions that frequently occur in genetic, acquired, and aging muscle diseases (Gorio et al., 1983). Previous and more recent studies increasingly suggest important feed forward and feedback mechanisms between the different cell types that mutually affect cell behavior (Carnio et al., 2014; Wu et al., 2015; Khan et al., 2016; Lee et al., 2017; Dobrowolny et al., 2018). So far, synoptic consideration of pathophysiological processes on the entire musculoskeletal organ has been severely hampered by two main factors. First, the mentioned mono-innervation of muscle fibers in combination with the extreme discrepancy between small NMJ size and large muscle fiber dimensions leads to an unequal distribution of these components in the organ; therefore, effects seen in one part of the muscle might not occur in others. Second, from a technical point of view, optical tissue clearing methods that would allow for such analysis of NMJs in their whole mount context have been lacking so far.

Introduced by Chung et al., CLARITY (Clear Lipid-exchanged Acrylamide-hybridized Rigid Imaging/Immunostaining/*in situ* hybridization-compatible Tissue-hydrogel) is one of the many new tissue clearing methods available and has gained a great deal of attention due to its robustness and compatibility with many different stainings (Chung and Deisseroth, 2013; Chung et al., 2013). This protocol and its variations (Lee et al., 2014; Tomer et al., 2014; Yang et al., 2014; Kim et al., 2015; Kleffel et al., 2016; Greenbaum et al., 2017; Du et al., 2018; Wang et al., 2018) address Refractive Index (RI) heterogeneity by first embedding the tissue in an acrylamide/bis-acrylamide based hydrogel. In addition to increasing tissue stability and porosity, this stabilizes the RI across the tissue from the estimated *n* = 1.50 of dry tissue to *n* = 1.457. Lipids are then drawn out of the embedded samples via active clearing in an electrophoresis chamber that applies a current and a continual stream of SDS over the tissue. This process increases the homogeneity of the RI throughout the sample even further, since lipids tend to have varying RIs and can increase light scattering when imaging deep into tissue. Even though this is a very promising method, Milgroom et al found it was incompatible with α-bungarotoxin (BGT) (Milgroom and Ralston, 2016), the most widely used postsynaptic NMJ marker, which labels nicotinic acetylcholine receptors (AChRs) with unmatched specificity. Their hypothesis was that the additional cross-linking and fixation prevented access of the toxin to the acetylcholine receptors (AChR). This incompatibility was further validated by Zhang et al., who found that even a modified passive CLARITY method resulted in the absence of BGT signals and appears to be very sensitive to standard optical clearing procedures (Zhang et al., 2018). Another study did report the presence of BGT fluorescence signals with the use of *in vivo* injected BGT in combination with a modified organic-solvent clearing protocol based on 3DISCO (Chen et al., 2016). Nonetheless, the combination of fluorophore compatibility/stability, tissue shrinkage, and the fact that *in vivo* injection of BGT hampers *post-hoc* stainings make this protocol and other organic solvent-based methods less than ideal for most applications.

Here, we address many of these issues by introducing a new optical tissue clearing protocol that is based on aldehyde fixation and hydrogel embedding. This robust protocol enables transparency of samples with a thickness >700 μm and is compatible with mouse diaphragm as well as EDL muscles. Additionally, it presents long-term fluorophore stability of NMJ staining in mouse skeletal muscle whole mounts.

## Materials and Methods

### Animals and Sample Preparation

In the current study, adult C57BL/10J, and BL10/JMDX mice were used. Animals were maintained in a local animal facility and their use and care were approved by German authorities according to EC directive 2010/63. For all experiments, adult mice were euthanized by cervical dislocation. Either whole hind limbs or just EDL muscles as well as diaphragm muscles were freshly dissected. Samples were then immediately immersed in 4% PFA/1x PBS and incubated for a minimum of 24 h on a roller mixer at 4°C.

### MYOCLEAR

A detailed protocol including reagent and equipment lists, photos of custom-made devices, and troubleshooting can be found in the Supplementary Methods section. Briefly, muscles were either freshly dissected or taken from PFA fixed mouse muscles. However, we recommend dissecting muscles from PFA fixed specimens since this tends to drastically reduce accidental damage to the tissue. Then, 100 mg of VA-044 initiator (final concentration 0.25%) and 40 ml of freshly prepared hydrogel monomer solution (A4P0) were added to 50 ml light resistant Falcon tubes, briefly hand mixed, and kept on ice to prevent premature polymerization. One muscle was then placed in each falcon tube and incubated on a roller mixer for 5 days at 4°C. After, muscles were degassed for 1 h via a custom-built degassing apparatus which allowed nitrogen to bubble over the samples (see Supplementary Methods section for photograph). The caps of the Falcon tubes were then loosely placed back on and the tubes transferred to an air tight desiccator where they were vacuumed under a 90 kPa negative pressure for an additional hour in order to purge any remaining oxygen from the sample. The desiccator was then flushed with nitrogen, Falcon tube caps tightened, and placed in a hot water bath at 37°C for 4 h with shaking for polymerization. Samples were then removed from the Falcon tube and excess hydrogel removed by washing samples with 1x PTwH overnight on a roller mixer at room temperature. It is important to note that in lieu of using a desiccator and hot water bath, we found that using Life Canvas's EasyGel system resulted in comparable results and made sample handling simpler and easier. However, the custom-built nitrogen bubbling apparatus was still needed to ensure uniform hydrogel polymerization.

For NMJ plus nuclei labeling, samples were stained as follows, inspired by the iDISCO staining protocol (Renier et al., 2014): Samples were washed in 1x PTwH with solution changes every hour for 2 h. After washing, samples were incubated in blocking and permeabilization solution (BnP) with shaking at 37°C for 48 h. Then, the BnP solution was replaced with 1 ml of fresh BnP solution, the dyes added [BGT-AF647 (1:200), BGT-AF555 (1:200), and/or DRAQ5 (1:300)], and allowed to incubate for 5 days at 37°C with shaking. After, samples were thoroughly washed in 1x PTwH with solution changes every 10 min, 15 min, 30 min, 1 h, and then every 2 h for a minimum of 2 days. The detergent was then removed by washing samples in distilled water for 4–8 h with frequent solution changes. Lastly, samples were incubated in 88% glycerol at room temperature for a minimum of 24 h for RI matching and long-term storage. Additionally, it was found that samples were stable for many months when stored in this manner. For indirect immunofluorescence staining, samples were processed as described above, with modifications as detailed in the Supplementary Methods. A list of primary and secondary antibodies and their dilutions can also be found there.

### X-CLARITY

For all samples stained after active clearing, a Biozym X-CLARITY protocol was followed, excluding the perfusion step. This is available for download from their website, https://www.biozym.com/. Briefly, EDL muscles were dissected from PFA fixed hind limbs and washed for 3 h in PBS at 4°C. Samples were then transferred to 50 ml light resistant falcon tubes containing 40 ml of freshly prepared 4% PFA/A4P0 monomer solution and 100 mg of VA-044 initiator, followed by a 5-day incubation on a roller mixer at 4°C. Then, samples were degassed via partial vacuum for 1 h, flushed with nitrogen, and incubated at 37°C for 3 h in a hot water bath to induce polymerization. Afterwards, samples were washed for 1–2 h in 1x PBS on a roller mixer at RT to remove excess hydrogel, transferred to an X-Clarity brain slice tissue holder, and lowered into the X-Clarity ETC chamber, where they were cleared for 3 h with 4% SDS buffer at a flow rate of 30 rpm; temperature: 37°C; current: 1.5 A. After, samples were thoroughly washed in PBST at 37°C for 24 h, then stored at 4°C in 1x PBS. For staining, samples were processed following the immunostaining section described in the Biozym protocol, with a dilution factor of 1:200 and 1:500 for BGT-AF647 and Wheat Germ Agglutinin CF488 conjugate (WGA-488; Biotium), respectively. Lastly, prepared samples were kept in 88% glycerol for storage and imaging.

### Active and Passive CLARITY

EDL muscles were processed following the MYOCLEAR protocol described above, followed by either active or passive clearing for 24 h in the X-CLARITY ETC tissue clearing system. Here, a steady flow of 4% SDS at a rate of 30 rpm was applied to the samples and the current either left off for passive clearing or adjusted according to the experiment for active clearing, with the maximum temperature recorded at the end of each run, see Supplementary Methods section. For experiments that addressed the effect refixation would have on preserving BGT fluorescence, samples were stained, incubated in 4% PFA for 24 h at 4°C, and then cleared. Lastly, all samples were stored and mounted in 88% Glycerol for imaging and kept at room temperature.

### Microscopy

Single stack acquisitions were imaged using a Leica Microsystems TCS SP2 equipped with a Leica Microsystems HC PL AP0 20x/0.75 IMM CORE CS2 objective, Leica confocal software version 2.61, a KrAr laser (488 nm, 514 nm), a diode-pumped laser (561 nm), and a HeNe laser (633 nm). For tile scans, an upright Leica Microsystems TCS SP8 equipped with LAS X software, a 488 nm laser, a 561 nm laser, a 633 nm laser, and Leica Microsystems clarity objective HC FLUOTAR L 25x/1.00 IMM (ne = 1.457) was used. 3D imaging of whole mount muscle samples was performed in 88% glycerol immersion using 6-cm round plastic dishes. Muscles were fixed by surgical thread knotted around the distal and proximal tendons. Visualization worked best after at least 24 h of temperature adjustment of the sample in the microscope room. During this period, the sample was kept in the dark.

### Fiber Number and Image Analysis, Figure Preparation, and Statistics

Transversal EDL cryo-sections (15-μm thick) from C57BL/10J mice were stained with WGA-488 (1:1,000 dilution in 2% BSA/PBS) for 15 min at RT to outline muscle fibers. After washing and embedding in Mowiol, sections were imaged with an inverted Leica SP8 microscope. After acquisition, all images were electronically processed using either Leica Microsystems LAS X core or ImageJ software. Signal-to-Noise-Ratio (SNR) measurements were done in ImageJ. Here, NMJs were segmented and mean intensities of the NMJs and standard deviation (SD) of adjacent fiber background regions were measured. The ratio of NMJ intensities vs. background SD was determined as SNR for each synapse. Numbers displayed in the text indicate the average of several SNR values per sample. For quantitative analysis of NMJ and fiber numbers, position of all observed NMJs / fibers was completed using the multi-point tool of ImageJ. This determined the xyz-position of the center of each NMJ / fiber. For analysis of critical morphological parameters of NMJs according to Jones et al. (2016), five square ROIs, each 500 × 500 μm, were selected per muscle. Then, all en face NMJs per ROI were manually thresholded and segmented using the magic wand tool in ImageJ. Then, area, perimeter and bounding rectangle diagonal were measured for every segmented NMJ. The number of AChR clusters per NMJs was counted manually. The diagonal of the bounding rectangle was calculated from the bounding rectangle sides while the fragmentation index was determined using the term: fragmentation index = 1–[1/(number of AChR clusters)]. Spectral un-mixing (Zimmermann et al., 2002) used the ImageJ plugin SpectralUnmixing (https://imagej.nih.gov/ij/plugins/spectral-unmixing.html). All figures were assembled using Adobe Illustrator. Mean values and standard deviations were calculated in Microsoft Excel. Normal distribution and homo/heteroscedasticity of data were probed using Kolmogorov-Smirnov test and *F*-test, respectively. According to these results and the type of data, statistical significance was evaluated using either one-way Analysis of Variance (ANOVA) with Tukey's *post-hoc* test, unpaired two-tailed *t*-test, or Kruskal-Wallis test. Bar graphs are presented as mean ± SD. *P*-values were indicated as ^*^(*p* < 0.05), ^**^(*p* ≤ 0.01), ^***^(*p* ≤ 0.001), or ^****^(*p* ≤ 0.0001). *P* ≥ 0.05 was considered not significant.

## Results

### An SDS-Free Hydrogel-Based Clearing Protocol Retains NMJ Staining

Previous attempts of optically clearing whole skeletal muscles using hydrogel-based, CLARITY-derived protocols yielded good tissue transparency but led to a quantitative loss of BGT fluorescence. To address possible reasons for this, we tested several variations of the procedure that was previously described by Milgroom and Ralston (2016). Samples were PFA fixed, embedded in hydrogel, and then stained with BGT-AF647 for NMJ labeling. Once stained, muscles were thoroughly washed for a minimum of 2 days, incubated in 88% glycerol overnight, and imaged to check for BGT-AF647 fluorescence. Notably, BGT-AF647 fluorescence was observed in all hydrogel-embedded samples (Supplementary Figure 1A, left panels). After initial imaging, glycerol was removed from the samples by washing for 24 h in PTwH at RT and were then actively cleared using an X-CLARITY tissue clearing system. A variety of settings, including different electrophoresis strengths, the addition of PFA fixation after BGT-AF647 staining, as well as a passive CLARITY protocol using a constant flow of SDS with no current, were tested. These modifications resulted in a reduced SNR of NMJ labeling in the cleared tissue (Supplementary Figure 1A, see lower left angles in panels for SNR values); samples that were post-fixed with PFA before clearing were less affected. However, also these re-fixed samples exhibited a large decrease in fluorescence intensity of the BGT-AF647 staining and were still far from acceptable quality. Thus, supporting the findings of previous studies which state the incompatibility of CLARITY-based protocols with BGT-NMJ staining.

The next step was to determine the effects of SDS on BGT staining: whether it washed out the membrane bound AChRs, simply quenched the fluorophore, or denatured the AChRs to the point BGT would not be able to bind. Samples were processed following the X-CLARITY protocol. Briefly, samples were PFA fixed, hydrogel embedded, actively cleared using an X-CLARITY machine, stained with either WGA-488 or BGT-AF647, and incubated in 88% glycerol for imaging. It was found here that staining the samples post-clearing with BGT-AF647 continued to result in an absence of NMJ signals, data not shown. However, NMJs in total were not destroyed by these techniques. Indeed, the lectin WGA could nicely identify the proteoglycan-rich ECM at the NMJs (Supplementary Figures 1B,C, see arrowheads in Supplementary Figure 1C for some examples of NMJs) besides other structures, such as blood vessels. Therefore, it might be assumed that SDS caused either quenching of the fluorophores or that it denatured the AChRs. The latter would, in turn, release BGT-AF647 from the AChR for samples stained before clearing and impede BGT-AF647 from binding altogether for samples stained post-clearing. With this in mind, SDS was excluded from all other experiments due to its role as a potential risk factor for the maintenance of BGT binding sites on NMJs.

**Figure 1:**
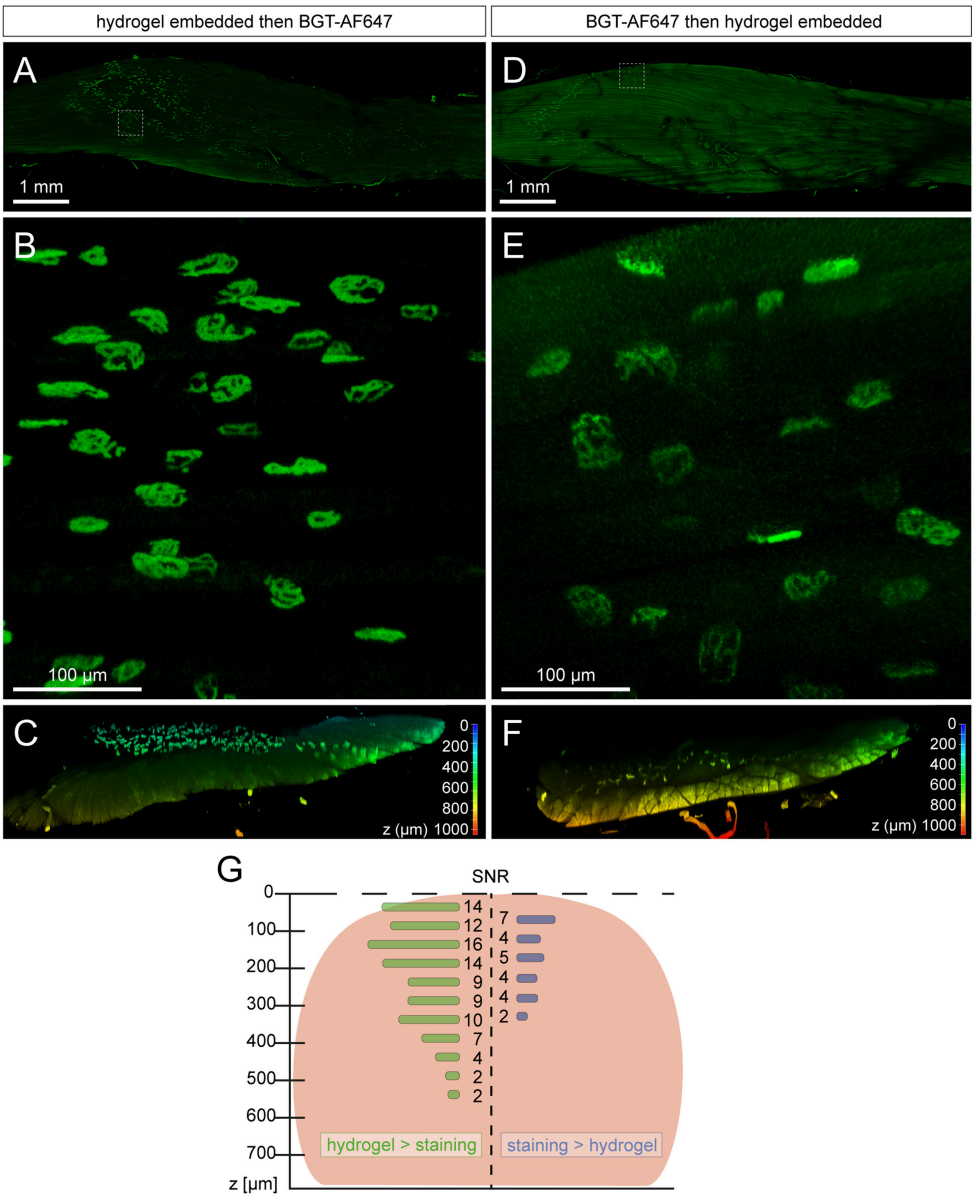
Sequence of staining and hydrogel embedding affects overall imaging depth and quality of muscle samples. All samples were imaged in 3D using a Leica SP8 confocal microscope and images were processed with Leica LAS X software. **(A–C)** Mouse EDL muscle was PFA-fixed, hydrogel embedded, stained with BGT-AF647, and then RI matched in 88% glycerol before imaging. **(D–F)** Mouse EDL was PFA-fixed, stained with BGT-AF647, hydrogel embedded, and then RI matched in 88% glycerol before imaging. **(A,D)** depict overviews of the whole mouse EDL muscles with the boxed region representing zooms shown in **(B,E)**. **(C,F)** portray cross sections cropped from the center of the EDL imaging data and depth-coded on the z-axis in order to visualize imaging depth and quality for both methods. **(G)** Graphical display of NMJ-signal SNRs in correspondence to muscle tissue depth and staining / clearing order. Muscle tissue extension in the central muscle region is depicted in the background as reddish round shape. Mean SNR values are shown as horizontal bars with corresponding numbers next to it. Left and right halves correspond to muscles shown in **(C,F)**, respectively.

**Figure 2:**
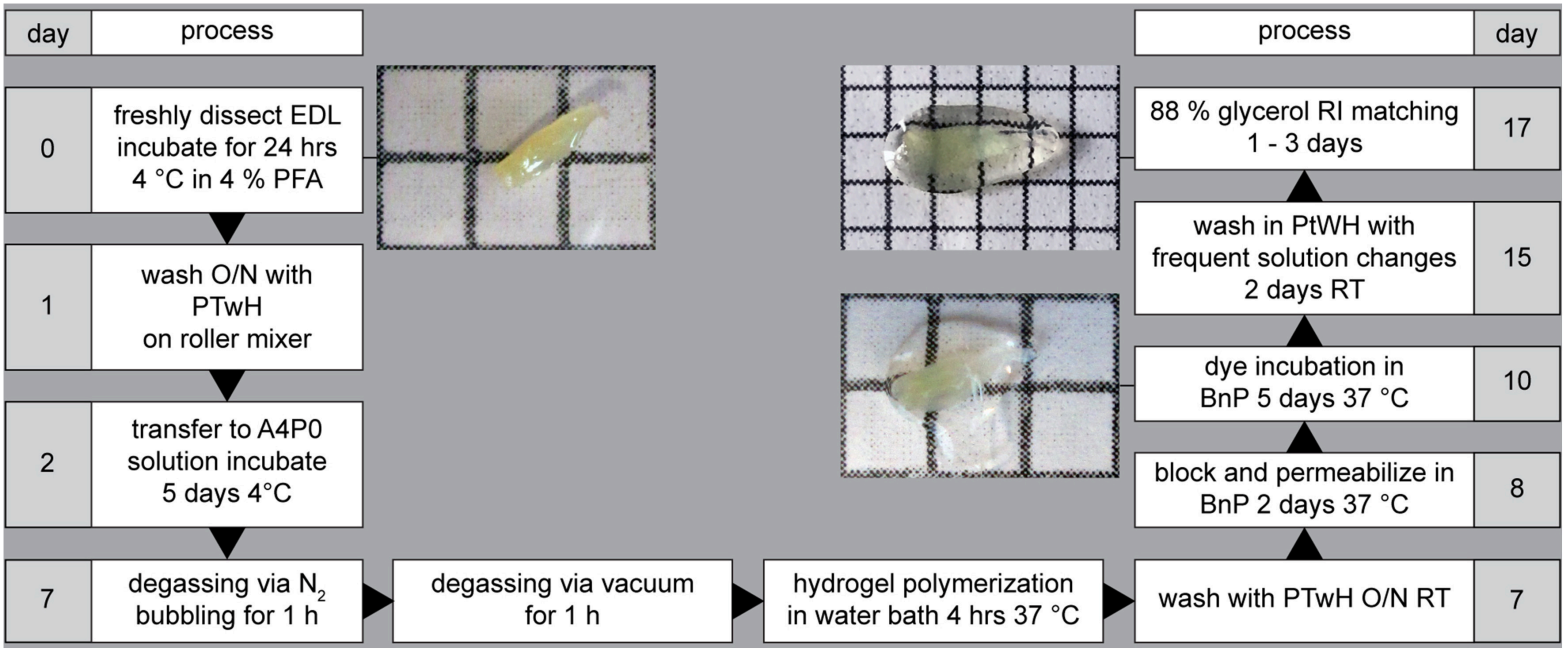
Overview of the MYOCLEAR protocol. MYOCLEAR can be divided into three major stages: hydrogel embedding (days 2–7), staining (days 8–15), and RI matching (days 16–17). The panel gives a graphical overview on the detailed descriptions found in Materials and Methods and Supplementary Methods sections. Photographs next to processing days 0, 10, and 17 show the appearance of EDL muscles at the start of the clearing protocol, before staining, and upon RI matching, respectively.

With the recent introduction of a new free-of-acrylamide SDS based tissue clearing protocol (Xu et al., 2017), the necessity of the embedded hydrogel and its effect on tissue needed to be explored. In theory, the embedded hydrogel not only homogenizes the RI throughout the sample but also increases the porosity of it; resulting in better penetration and uniformity of staining (Chung and Deisseroth, 2013; Chung et al., 2013). To test the effects of the hydrogel, samples were PFA fixed and either embedded in hydrogel, then stained with BGT-AF647 or vice versa. Both being compared in Figure 1, we found that staining samples after hydrogel embedding led to a better SNR (Figure 1G, Supplementary Figure 2) and increased the overall imaging depth of the sample (Figure 1G, Supplementary Figure 2). This confirmed the need of embedding samples with hydrogel and resulted in the final protocol termed MYOCLEAR. This method represents a passive hydrogel-based clearing method for the visualization of NMJs in fixed mouse muscles and is summarized in Figure 2.

### Muscle Fibers, Nuclei, and NMJs Can Be Visualized by Virtue of Green/Red Autofluorescence and Spectral Unmixing of Near-Infrared Fluorescence Signals

**Figure 3:**
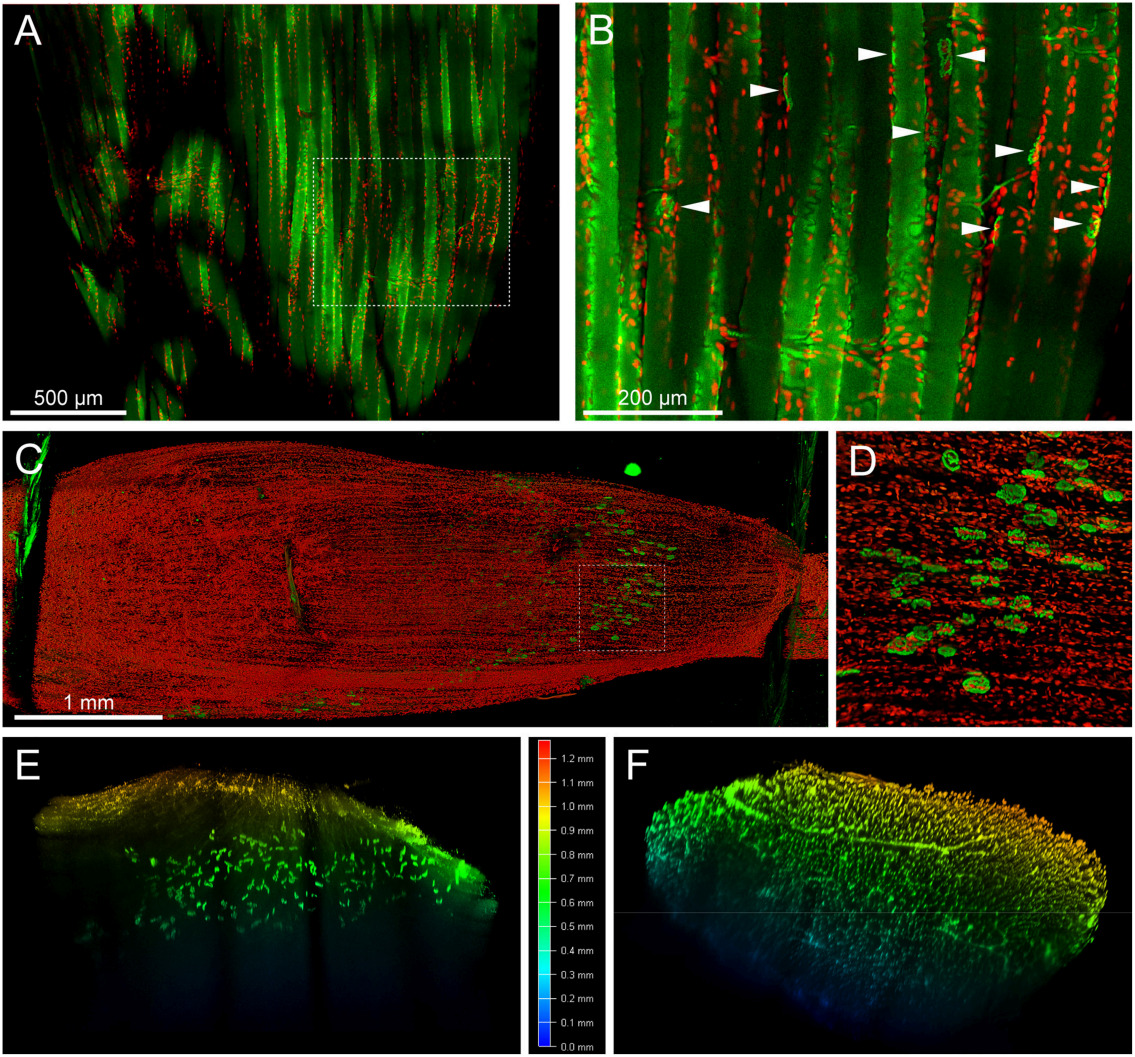
MYOCLEAR enables imaging of muscle fibers, myonuclei, and NMJs by using red autofluorescence and spectral unmixing of far-red wavelengths dyes. **(A,B)** Mouse EDL was processed via the MYOCLEAR protocol and stained with BGT-AF555 and Draq5. **(A)** depicts a confocal section of the EDL, with **(B)** representing a zoom of the boxed region. Strong autofluorescence of the tissue in the AF555 channel (green) resulted in a poor SNR for NMJ detection (some NMJs are highlighted in **B**, arrowheads). In contrast, the near-infrared dye Draq5 displayed crisp and clear nuclei. **(C–F)** Mouse EDL muscle was processed via the MYOCLEAR protocol and stained with BGT-AF647 and Draq5. In order to overcome the auto-fluorescence shown in this figure, the emission windows for each dye were adjusted according to their peak values and acquired separately using the same 633-nm excitation laser on a SP8 confocal microscope. The images were processed using Leica LAS X software and spectrally un-mixed in ImageJ. **(C)** Maximum-z projection of the whole EDL before applying spectral un-mixing. Draq5, red; BGT-AF647, green. Green autofluorescence of the thread keeping the muscle in place for imaging is visible at the proximal and distal ends of the muscle. **(D)** Zoom view of the boxed region in **(A)**. **(E,F)** Z-axis depth coding for signals of BGT-AF647 **(E)** and Draq5 **(F)** shown as cross sections after spectral un-mixing.

Number and position of myonuclei can serve as relevant parameters in muscle research. Specifically, detection of centro-nucleated fibers in diseased and regenerating muscle or analyzing the presence of fundamental subsynaptic nuclei at NMJs would need determination of these parameters. Thus, we sought to use Draq5, a near-infrared nuclear dye, in combination with a red-fluorescent BGT-AlexaFluor555 conjugate on MYOCLEAR-treated EDL muscles. As shown in red in Figures 3A,B, nuclei were well-stained and visible in the Draq5 channel. Conversely, NMJs, indicated by arrowheads in Figure 3B, were barely visible due to massive, PFA-induced autofluorescence (both shown in green). Consequently, this made the quality of these results inadequate for analysis. Furthermore, the intense level of autofluorescence was also observed in the 500–550 nm wavelength range when samples were stained with BGT-AlexaFluor488, data not shown.

**Figure 4:**
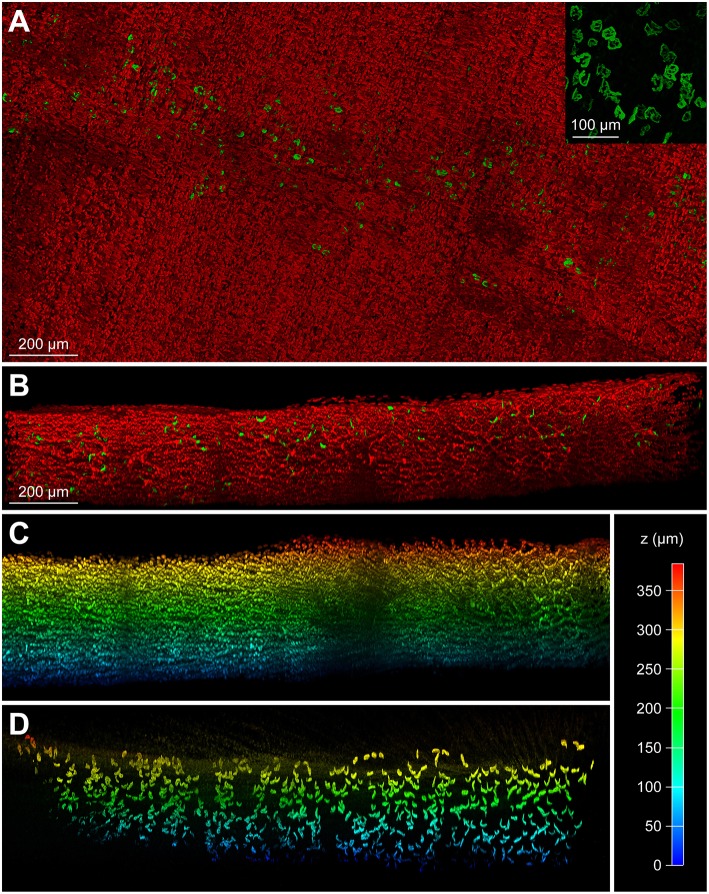
MYOCLEAR is functional with adult mouse diaphragm muscle. Adult mouse diaphragm muscles were processed using the MYOCLEAR protocol and stained with Draq5 and BGT-AF647. **(A)** Maximum-z projection of a representative tile stack showing fluorescence signals of BGT and Draq5 in green and red, respectively. Note, that NMJs are not fragmented but partially covered by myonuclei. This is evident in the insert, which shows only BGT signals of a small region. **(B)** Side view to show depth extension of fluorescence signals. The entire depth of around 500 μm of the diaphragm became transparent. **(C,D)** Depth coded side views of nuclear **(C)** and NMJ signals **(D)**. Pseudocolor code is explained on the right side.

To mitigate the autofluorescence-induced limitation observed in short-wavelength fluorescence channels, we utilized two slightly spectrally separated near-infrared dyes, BGT-AF647 (maxima of excitation and emission, 650 and 665 nm, respectively) and Draq5 (maxima of excitation and emission, 646 and 681 nm, respectively). Muscles were PFA fixed, hydrogel embedded, co-stained with the dyes mentioned above, incubated in 88% glycerol, and then imaged using a 633 nm wavelength excitation laser for both. Each dye was acquired separately and their emission detection windows adjusted to 643–679 nm and 685–778 nm for BGT-AF647 and Draq5, respectively. Figure 3C depicts a maximum-z projection of an EDL-whole mount scanned over a thickness of 1.2 mm. In addition, Figure 3D and Supplementary Video S1 show a zoom view of the boxed region and a rotation of the data projection, respectively. Nuclei and NMJs could be clearly distinguished. An additional spectral un-mixing step (see chapter Fiber number and image analysis, figure preparation, and statistics) was then added for more accurate segmentation results. Figures 3E,F show depth-coded side views of BGT-AF647 and Draq5, respectively, and reveal a good signal penetration for both over a depth range of about 1 mm. Figure 3F displays some elongated structures, which likely represent blood vessels traversing the muscle. To assess the compatibility of MYOCLEAR with muscle types other than EDL, we applied the protocol to adult mouse diaphragm. Confocal analysis revealed that the procedure was good to achieve complete penetration of diaphragm muscle in z (Figures 4A–D). The insert in Figure 4A shows that NMJs were well-preserved in these samples. The apparent fragmentation in the large overview in Figure 4A is due to nuclei partially covering many synapses.

### Whole Mount Analysis Detects Local Heterogeneity of NMJ Fragmentation Index in mdx Muscle

**Figure 5:**
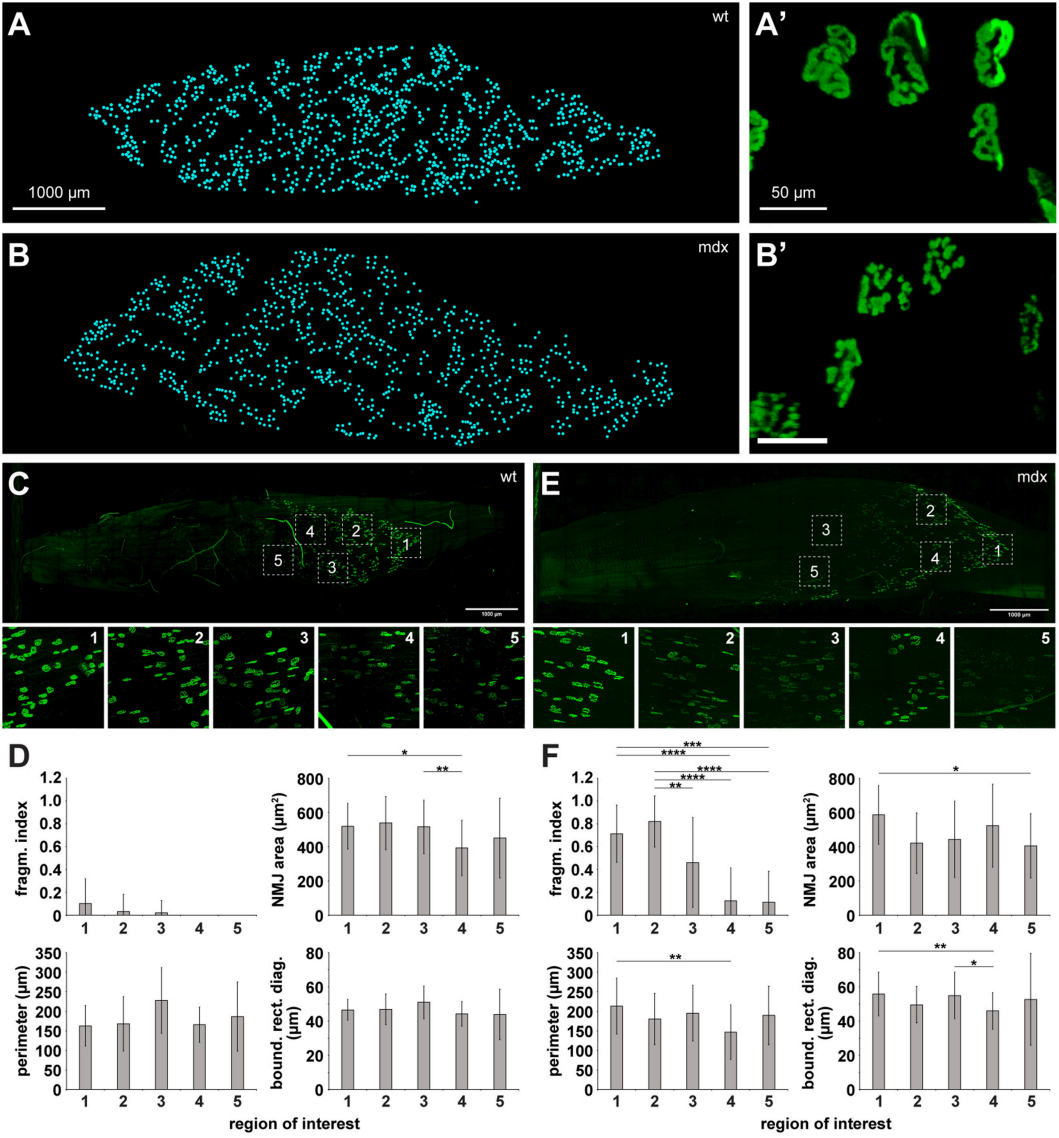
Analysis of whole mount NMJ morphology and quantification of NMJ numbers is enabled in wildtype and mdx muscles upon clearing. EDL muscles from wildtype **(A,A',C,D)** and dystrophic mdx mice **(B,B',E,F)** were processed using the MYOCLEAR protocol and stained with BGT-AF647. **(A,B)** Maximum-z projections of all NMJs detected by hand segmentation. Each cyan spot represents a single NMJ. **(A',B')** High-power images of some representative NMJs from each muscle shown in **(A,B)**. **(C,E)** Upper panels, maximum-z projections of representative muscles showing BGT-staining signals. Lower panels, high power display of ROIs 1–5 in corresponding upper panel. **(D,F)** Quantitative analysis of key morphological parameters: area, fragmentation index, perimeter, and bounding rectangle diagonal of NMJs. Depicted are mean ± SD for all en face NMJs detected as a function of ROI number. ^*^p < 0.05, ^**^p ≤ 0.01, ^***^p ≤ 0.001, ^****^p ≤ 0.0001.

Wildtype skeletal muscle, as tested so far, is characterized by extremely homogeneous tissue composition. Conversely, diseased muscles might exhibit large amounts of fibrosis, fatty tissue, immune cell aggregates, or other changes that can affect the optical characteristics of muscle tissue and their transparency after clearing. Thus, we looked at muscles from wildtype and dystrophic mdx mice. The latter are characterized by extensive fibrosis (Piñol-Jurado et al., 2018) and fragmented NMJs (Lyons and Slater, 1991; Röder et al., 2012). We found that applying MYOCLEAR to wildtype and mdx muscles resulted in data of comparable quality. As expected, NMJ structure was clearly different between the two; with wildtype NMJs displaying coherent pretzel-like structures and mdx NMJs demonstrating fragmented morphology. Next, whole mount imaging data was acquired for some wildtype and mdx mouse EDLs that were processed with MYOCLEAR and stained with BGT-AF647. The numbers of visible NMJs were then quantified by hand using the multi-point tool in ImageJ. Representative muscles are depicted in Figures 5A,B which illustrates the ability of this protocol to detect hundreds of NMJs. In quantitative terms, 1082.3 ± 29.5 and 1019.5 ± 14.8 (each mean ± SD) NMJs were counted in wildtype and mdx muscles, respectively. Figures 5A',B' show representative high-power images of a few NMJs from each of the corresponding muscles. These panels demonstrate the normal, pretzel-like structure of NMJs in wildtype (Figure 5A') as compared to the fragmented appearance in the mdx muscle (Figure 5B'). An important advantage of whole mounts should be that heterogeneity of objects or effects of treatments within the entire organ can be better observed than in individual tissue sections. To assess this point, key morphological parameters of NMJs from different ROIs of both, wildtype and mdx muscles were determined and compared. Therefore, a subset of criteria recently introduced by Jones et al. was applied (Jones et al., 2016). In detail, area, fragmentation index, perimeter, and bounding rectangle diagonal of NMJs from five different ROIs (Figures 5C–F) per muscle were determined. NMJ areas, perimeters, and bounding rectangle diagonals were similar between all ROIs of a given muscle and also apparently not different between wildtype and mdx. Conversely, NMJ fragmentation index was higher in mdx than in wildtype and, furthermore, varied considerably within a given mdx muscle.

### VAChT Antibody Staining Confirms Integrity of NMJ Presynaptic Apparatus Upon MYOCLEAR

**Figure 6:**
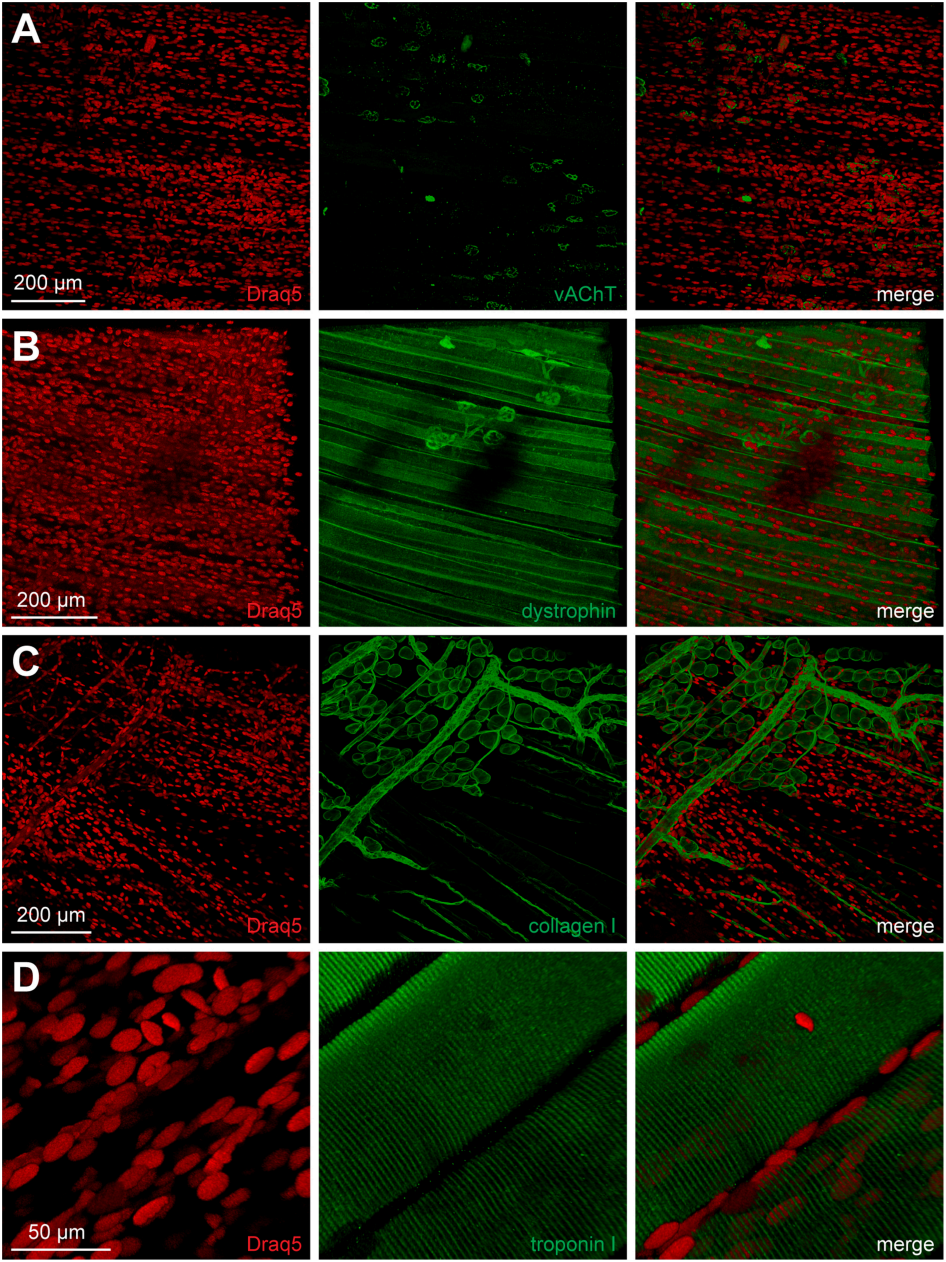
Integrity of NMJ presynapse and other muscle structures is maintained upon clearing. Adult mouse EDL muscles were processed using the MYOCLEAR protocol and co-stained with Draq5 and antibodies against either NMJ presynapse (**A**, vAChT), sarcolemma (**B**, dystrophin), ECM (**C**, collagen I), or sarcomere (**D**, troponin I). Images show maximum-z projections of confocal z-stacks with an interplane interval of 2 μm and depths from muscle surface of 466, 665, 104, and 214 μm for **(A–D)**, respectively.

BGT-AF647 and lectin staining data suggested that the NMJ ECM and postsynaptic apparatus remained intact during MYOCLEAR processing. To address presynaptic integrity and the amenability of the clearing protocol for immunofluorescence staining, we first processed EDL muscles with the MYOCLEAR protocol and then stained nuclei and the presynaptic NMJ marker protein vAChT using Draq5 and anti-vAChT antibody, respectively. As depicted in Figure 6A, the obtained antibody staining was concentrated in the NMJ regions, although considerable noisy signals were also observed outside the synaptic regions. Yet, NMJ presynapses showed normal coherent appearance, demonstrating that the clearing procedure did not affect this part either. The general integrity of the major muscle compartment was also confirmed by immunostaining with a few additional antibodies. Nicely, dystrophin outlined muscle fibers and was also enriched in the NMJ regions as expected (Figure 6B). Further labeling with collagen I antibody showed the distribution of large blood vessels, capillaries, and fascia cells (Figure 6C). Finally, immunostaining of troponin I retrieved the regular pattern of sarcomeric striations (Figure 6D).

## Discussion

Although previous attempts of optically clearing whole skeletal muscles using hydrogel-based, CLARITY-derived protocols yielded sufficient muscle tissue transparency, they led to a quantitative loss of BGT fluorescence. This was true for both, active (Milgroom and Ralston, 2016) and passive CLARITY protocols (Zhang et al., 2018). The interpretation in these studies was that hydrogel cross-linking coupled with PFA fixation prevented access of the toxin to AChRs. Conversely, we present a hydrogel and PFA based skeletal muscle clearing protocol that nicely retains BGT-based NMJ staining and exhibits a good light penetration of approximately 1,000 μm in mouse EDL muscle. This suggests, that SDS rather than hydrogel or PFA led to the loss of BGT staining. For simplicity, this method was termed MYOCLEAR. It allows for the analysis of whole mount NMJ counts in correlation to myonuclei analysis and is also compatible with diaphragm, as well as with other dyes, including lectins and antibodies. Given that the protocol is based on an initial PFA fixation step, it should be compatible with easy handling and material exchange.

Permutation of the protocol settings revealed that staining and image quality were superior if BGT labeling occurred after PFA fixation and hydrogel embedding. Since both methods (i.e., staining before or after hydrogel embedding) received ample washing steps, it is safe to assume the hydrogel either supports BGT-AF647 in a way that it can reach its target more accurately or aids in washing out unspecific BGT-AF647 signals. The present protocol presents strong autofluorescence in the blue to red fluorescence wavelength range, which is most likely due to PFA-fixation induced chromophore formation (Baschong et al., 2001). In the near infrared range, though, the autofluorescence issue was not present and thus AlexaFluo647 and Draq5 dyes worked well. Although the autofluorescence obtained in the green and red fluorescence channels might be considered a limitation of the MYOCLEAR protocol, it can also be rather useful for tracking individual muscle fibers over their whole length. Additionally, it can be used to assess pathophysiologically relevant features, including centro-nucleated regions, fiber atrophy, fiber splitting, the occurrence of poly-innervation, and other general structural information.

Given that most standard available fluorescence microscopy systems exhibit excitation lasers typically up to 633 nm wavelength, the use of near infrared dyes compatible with MYOCLEAR is somewhat limited. To permit at least two different structures to be simultaneously marked, we used the slightly wavelength-shifted dyes AlexaFluor647 and Draq5 in combination with spectral unmixing (Zimmermann et al., 2002). Using hand segmentation of BGT-AF647 stained and cleared EDL wholemounts, slightly more than one thousand NMJs were identified in each muscle. According to literature, the amount of muscle fibers in an adult mouse EDL ranges from 758 to 1,147 (White et al., 2010; Bloemberg and Quadrilatero, 2012). To confirm this, we performed fiber counts from our own animals by analyzing cross sections of contralateral muscles. This revealed fiber numbers of 1,052 ± 42 (mean ± SD) per EDL muscle. Thus, taking into account that each muscle fiber in adult muscle is innervated by one single NMJ (Krause, 1863), this finding supports that the total number of NMJs obtained in our cleared muscles represents most, if not all, of the NMJs in the tissue. However, it must be stated that NMJs beyond a depth of about 700 to 800 μm from the surface were hardly above background and would render automated segmentation, volumetric, or in-depth morphological analyses of these synapses impossible. If such information is needed, it would be necessary to image muscles from both sides and eliminate the overlapping NMJs.

MYOCLEAR was found to retain morphological integrity of presynapse, synaptic cleft, and postsynapse. In many experimental paradigms, considerable regional heterogeneity of critical morphological parameters of the neuromuscular apparatus might be expected, such as for NMJ degeneration/regeneration in dystrophic mouse models (Haddix et al., 2018), terminal sprouting upon neurotoxin application (Wright et al., 2007; Duregotti et al., 2015), or in aging muscle (Valdez et al., 2010). Therefore, a more holistic picture of observed changes in the whole mount might yield relevant new insights. For wildtype and mdx EDL muscles, we addressed the principal applicability of our samples to study a subset of morphological criteria as recently proposed by Jones et al. (2016). This revealed differences with respect to the amplitude and regional heterogeneity of NMJ fragmentation index between wildtype and mdx, suggesting that whole mount analysis might serve as a valuable tool for future investigations of neuromuscular disorders. Certainly, immunofluorescence procedures will be a relevant asset to perform more in-depth analyses in this context. Thus, it was evaluated, whether the present clearing protocol is compatible with antibody staining. Indeed, immunostaining with all tested antibodies against presynapse (vAChT), sarcomere (troponin I), sarcolemma (dystrophin), and extracellular matrix (collagen I) yielded the expected staining patterns, although depth penetration still needs to be optimized. Apart from vAChT, which was visible beyond 500 μm deep in the tissue, the other markers were visible only for about 200–300 μm of depth. It will be necessary to examine if additional techniques for dye distribution, such as stochastic electrotransport or similar procedures (Kim et al., 2015; Nehrhoff et al., 2016) might solve the issue of penetration. Also, the limitation of MYOCLEAR to near-infrared dyes requests evaluation of further dye combinations that would be compatible with AF647 and Draq5. We tested secondary antibodies coupled to PE-Cy7, but were not successful due to low secondary antibody specificity.

In summary, future work will have to deal with skeletal muscle clearing protocols that are compatible with a wider spectral range to incorporate more than just two dyes in one sample as well as with reaching a higher depth penetration of antibodies. Furthermore, besides improving the MYOCLEAR protocol, an automated quantitative determination of NMJ numbers and characteristics, such as size and fragmentation, would likely be a major analytical request for cleared muscles. We have started to work on such automated detection algorithms, but they need further improvement before being valid. Though, Supplementary Video S2 shows a future prospect of advantages of such an approach. It depicts a 3D view of all NMJs detected in the muscle sample shown in Figure 5C. Yellow-coded NMJs were detected by auto segmentation, blue NMJs were detected by hand. It is evident that three-dimensional information gives a much more plastic view on the synapse band in this muscle.

## Ethics Statement

This study was carried out in accordance with the recommendations of EC directive 2010/63. The protocol was approved by the Regierungspräsidium Karlsruhe.

## Author Contributions

MW, MRi, TS, SH, and MRe performed the experiments and analyzed data. MW, MT, NG, MH, and RR planned experiments, contributed material, and wrote the paper.

### Conflict of Interest Statement

The authors declare that the research was conducted in the absence of any commercial or financial relationships that could be construed as a potential conflict of interest.
