# Supplementary material for: A Novel Optical Tissue Clearing Protocol for Mouse Skeletal Muscle to Visualize Endplates in Their Tissue Context
Source: Front Cell Neurosci. 2019 Feb 27;13:49. doi: 10.3389/fncel.2019.00049 (PMC6401545; doi:10.3389/fncel.2019.00049)
Supplement: Supplementary file 3 [file Data_Sheet_1.docx]

Supplementary Material

A Novel Optical Tissue Clearing Protocol for Mouse Skeletal Muscle to Visualize Endplates in Their Tissue Context

**Marion Patrick Ivey Williams, Matteo Rigon, Tatjana Straka, Sarah Janice Hörner, Norbert Gretz, Mathias Hafner, Markus Reischl, and Rüdiger Rudolf***

*** Correspondence:** Rüdiger Rudolf: r.rudolf@hs-mannheim.de

## Supplementary Methods

**Detailed MYOCLEAR protocol**

***PFA Fixation:***

1. Prepare fresh 4% PFA solution.

Caution: PFA solution must be fresh!

1. Freshly dissect whole hind limb or EDL muscles directly.

Note: we recommend taking the whole hind limb and then taking EDL muscles since this tends to decrease the chances of accidental damage to the muscle during dissection.

1. Transfer samples into an appropriate tube and add enough 4 % PFA to generously cover sample
2. Place tubes on a roller mixer at 4 °C for a maximum of 24 hrs.

- Caution: leaving samples in PFA for longer than the recommended time can cause over fixation, fragility, and degradation.

1. Remove samples from PFA and wash overnight with 1x PTwH on a roller mixer at room temperature to remove access PFA.

***Hydrogel Imbedding:***

Important: Keep all solutions cold throughout whole procedure. Failure to do so will lead to premature polymerization.

Before sample processing, prepare the following:

1. Gather one light resistant (brown or black) 50 ml Falcon tube per sample.
2. Weigh 100 mg of VA-044 activator into each falcon tube.
3. prepare 4 % acrylamide solution - enough for 40 ml per sample.

Caution: Must be prepared fresh. Additionally, do not use PBS in this step as the salts may interfere with the polymerization process.

1. add 40 ml of 4 % acrylamide to each falcon tube containing the VA-044 activator.
2. hand mix Falcon tubes until activator is in solution.
3. Add one sample per Falcon tube and incubate on a roller mixer for five days at 4 °C.

***Hydrogel polymerization:***

Important: Samples must be thoroughly degassed. The utmost care should be taken throughout the entire process to avoid reintroducing oxygen. Failure to do so will result in non-uniform polymerization and a decrease in imaging depth and quality.

***For polymerizing samples with a desiccator and hot water bath:***

1. For initial degassing, bubble nitrogen over samples for 1 hr.

Note: This was done using an in-house degassing chamber, see below. Samples should be kept cold during the process.


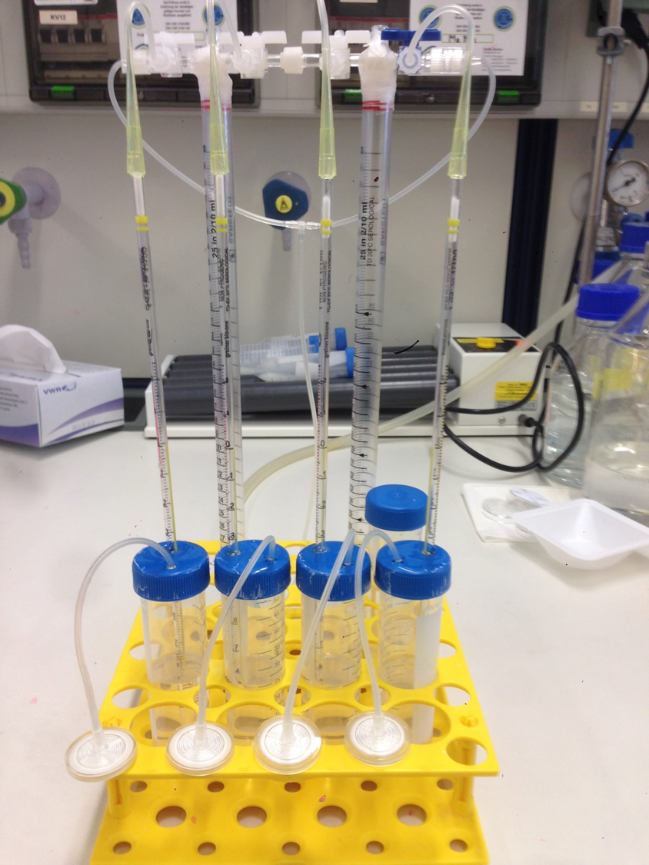


1. While removing samples from the bubbling chamber, keep nitrogen on and then tighten caps. This is to avoid oxygen from coming back into the Falcon tubes.
2. Transfer samples to a desiccator, loosen the caps on each sample, and vacuum the samples at 90 kPa.
3. Stop vacuum and flush with nitrogen.
4. Repeat vacuum and flush with nitrogen.
5. Vacuum once more and let run for 1 hr and then flush with nitrogen.
6. While continuing the flow of nitrogen, reach under vacuum hood and tighten the sample caps.

Caution: avoid exposing samples to oxygen during this time.

1. Transfer samples to a hot water bath and incubate samples at 37 °C for 4 hrs with shaking.
2. Remove Falcon tubes from the incubator and transfer samples to 2 ml Eppendorf tubes containing 2 ml of 1x PTwH.
3. Place Eppendorf tubes in light protected 50 ml Falcon tubes and wash at room temperature on a roller mixer overnight in order to wash off any excess hydrogel. Replace PTwH every so often for better results.

***For polymerizing samples with Life Canvas EasyGel system:***

1. Follow steps 1 – 2 described above for initial degassing via nitrogen bubbling.
2. Transfer samples to Life Canvas EasyGel system and vacuum for 1 hr at a negative pressure of 90 kPa.
3. Remove vacuum and flush samples with nitrogen.
4. Set heat to 37 °C and incubate for 4 hrs with shaking.
5. Follow steps 9 – 10 as described above to remove excess hydrogel.

***Staining protocol:***

PTwH = 1xPBS/0.5 %Tween-20 with 10 ug/ml heparin

1. Wash samples in 1x PTwH for 2 hrs with solution changes every hour.
2. Block and Permeabilize samples in BnP solution for two days on a roller mixer at 37 °C.

BnP solution for 100 ml:

- - 1. 10 ml 10x PBS
    2. 10 ml PTwH
    3. 500 µl Triton X-100
    4. 10 ml DMSO
    5. 6 g BSA
    6. 63.5 ml DI H_2_O

1. Replace BnP solution with 1 ml of fresh BnP.
2. Add dyes (or only primary antibodies) and incubate for five days at 37 °C on a roller mixer.

For co-staining samples: Both BGT-AF647 (1:200) and Draq5 (1:300) can be incubated together, as this will not affect staining performance. Though, it should be noted that this may not hold true when trying other dye combinations.

1. For antibody stainings: wash samples for 2 days in PTwH with frequent solution changes.
2. Antibody stainings continued: After washing for 2 days, transfer samples into BnP solution and add secondary antibodies and any dye/toxin stainings (DRAQ5 and BGT). Incubate for 5 days on a roller mixer at 37 °C.

For co-staining samples: same rules apply as stated above. It is important that if staining with an antibody and dye. The dyes are added during this incubation and not with the primary antibody.

1. Wash samples with PTwH solution for a minimum of two days with solution changes at every 10 min, 15 min, 30 min, 1 hr, and then every 2 hrs.

***Optical clearing and imaging:***

1. Wash stained samples in distilled water on a roller mixer at RT for a minimum of 6 hrs, with frequent solution changes. This is to wash out any detergent in the sample as it can interfere with imaging.
2. Transfer samples into an 88 % Glycerol solution and incubate on a roller mixer at room temperature for a minimum of 24 hrs before imaging.

Important: Extending this incubation time is recommended for troublesome samples since it creates a more homogenous RI throughout the sample. In addition, the refractive index of the Glycerol solution should be as close as possible to n = 1.457. This insures compatibility with Leica X HC FLUOTAR L 25x/1.00 IMM motCORR VISIR objective, as well as matching the hydrogel’s RI of n =1.457.

1. Mount samples in 88% glycerol and place on microscope for image acquisition. For this purpose, we used a custom-made chamber as shown below. This allows fixation of the muscle during image acquisition and sufficient access for the immersion objective.

Note: It is recommended to mount samples in the same 88 % glycerol that it was incubated in for RI matching. This drastically reduces the swirling effect caused by RI mismatches in the mounting solution.


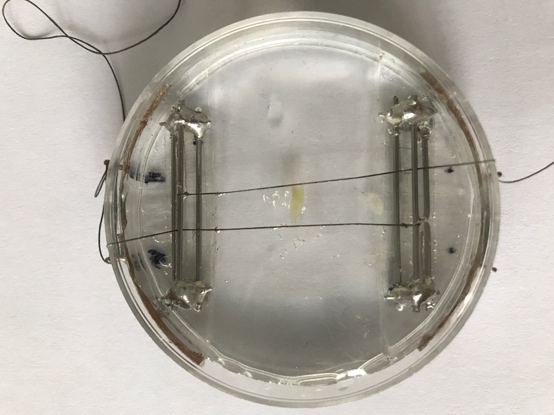

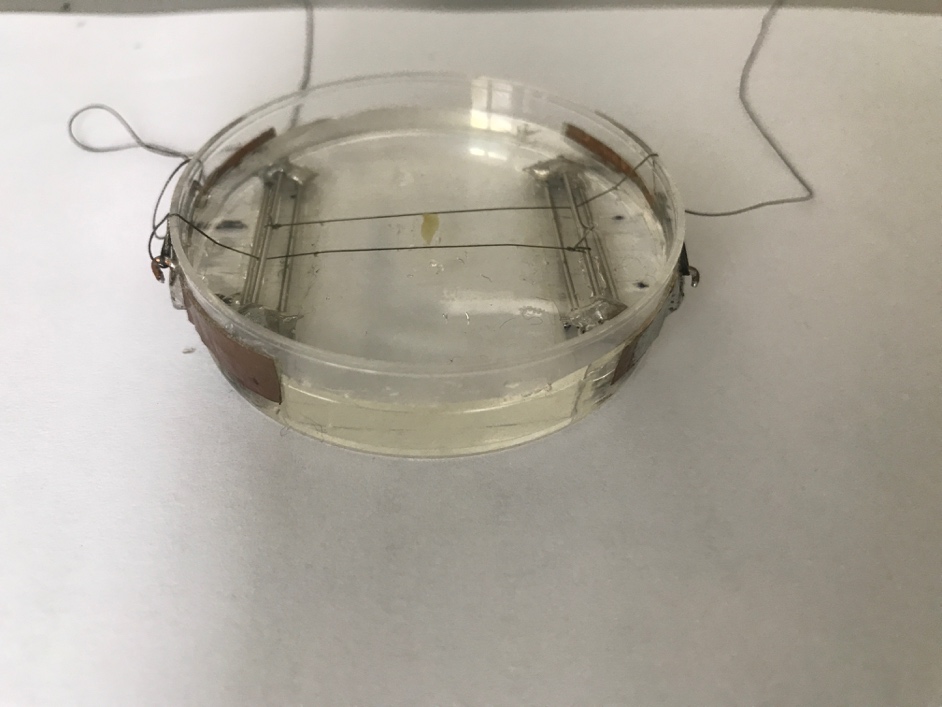


**List of used reagents**

| **Step** | **Solutions** | **Material** | **Company** | **Cat. Number** |
| --- | --- | --- | --- | --- |
| Sample fixation | 4 % Paraformaldehyde | PFA | Roth | 335,2 |
| Hydrogel monomer immersion | Hyrogel monomer solution (A4P0) | 40% acrylamide solution | BIO-RAD | 161-0140 |
|  |  | VA-044 (2,2'-Azobis [2-(2-imidazolin-22yl) propane] Dihydrochloride) | Wako | 011-19365 |
|  |  | Distilled water | in house |  |
| Passive and active clearing | Tissue clearing solution (4% SDS) | Sodium hydroxide (NAOH) | Sigma | 38227 |
|  |  | Boric acid | Applichem | A2940 |
|  |  | SDS | Roth | CN30.3 |
|  |  | Distilled water | in-house |  |
| Staining | Wash solution (1xPBS/0.5 % TritonX-100) | 1x PBS | in-house |  |
|  |  | TritonX-100 | Roth | 3051.4 |
|  | PTwH solution (1xPBS/0.5 % Tween-20 with 10 ug/ml heparin) | 1x PBS | in-house |  |
|  |  | Tween-20 | Sigma | P7949 |
|  |  | Heparin | Sigma | H3393 |
|  | Block and Permeabilize solution (BnP) (1xPBS/1x PTwH/0.5 % TritonX-100/ 10 % DMSO/6 % BSA) | 1x PBS | in-house |  |
|  |  | 1x PTwH | in-house |  |
|  |  | TritonX-100 | Roth | 3051.4 |
|  |  | DMSO | Roth | 5179.1 |
|  |  | BSA | PAA | K45 |
|  | BGT-AF647 (1:200) | BGT-AF647 | Invitrogen | B35450 |
|  | BGT-AF555 (1:200) | BGT-AF555 | Invitrogen | B35451 |
|  | Draq5 (1:300) | DRAQ5 | Thermo Scientific | 62251 |
|  | WGA-488 (1:500) | WGA-CF488 | Biotium | 29022 |
|  | rabbit anti-collagen I (1:50) | rabbit anti-collagen I | Rockland | 600-401-103-0.5 |
|  | rabbit anti-vAChT (1:50) | rabbit anti-vAChT | Synaptic Systems | 139 103 |
|  | rabbit anti-troponin I (1:25) | rabbit anti-troponin I | Cell Signalling | 4002 |
|  | rabbit anti-dystrophin (1:50, discontinued) | rabbit anti-dystrophin | MBL Int. Corp. | SM-3586-100, lot 11076 |
|  | anti-rabbit-AF647 (1:200) | donkey anti-rabbit-AF647 | Invitrogen | A21206 |
| Mount and storage | Glycerol (88%) | Distilled water | in house |  |
|  |  | >99.9% anhydrous glycerol | Kraft | 1736762 |

**List of used equipment**

| **Use** | **Product** | **Company** | **Model Number** |
| --- | --- | --- | --- |
| Hydrogel polymerization | Nitrogen Bubbler | in-house | see figure above |
|  | Desiccator | Bel-Art | F42027 |
|  | Vacuum pump | Welch | 2522c |
|  | water bath | Medingen | W 22 |
| Hydrogel polymerization with EasyGel system | Nitrogen Bubbler | in-house | see figure above |
|  | EasyGel | Life Canvas  Technologies | EasyGel |
|  | Vacuum pump | Welch | 2522c |
| ETC Chamber for active and passive CLARITY | ETC Chamber | Logos Biosystems | C10101 |
|  | ETC Chamber controller | Logos Biosystems | C10201 |
|  | Peristatic pump | Logos Biosystems | C10301 |
|  | Buffer reservoir | Logos Biosystems | C10401 |
|  | Tissue container | Logos Biosystems | C12001 |
|  | Mouse brain slice holder | Logos Biosystems | C12004 |
|  | Peristaltic pump tubing | Logos Biosystems | C12104 |
|  | Reservoir Cap with temperature probe | Logos Biosystems | C12102 |
| Imaging | TCS SP8 | Leica Microsystems | - |
|  | HC PL AP0 20x/0.75 IMM CORE CS2 objective | Leica Microsystems | - |
|  | HC FLUOTAR L 25x/1.00 IMM motCORR VISIR objective | Leica Microsystems |  |
|  | Mounting Dish | in-house | see figure above |
| Processing Software | Fiji (ImageJ) | NIH | - |
|  | LAS X | Leica Microsystems | - |

## Supplementary Figures

**Supplementary Figure 1.** **Standard active and passive CLARITY-derived methods lead to quantitative loss of BGT-AF647 staining but retain Lectin-mediated labelling of NMJ ECM.** (A) EDL whole mounts were PFA fixed, embedded in hydrogel, and stained with BGT-AF647. After overnight incubation in 88 % glycerol, BGT-AF647 fluorescence was checked (before clearing). Then, glycerol was washed out via PTwH followed by different clearing procedures as indicated on the left. Finally, all samples were again equilibrated in 88 % glycerol and BGT-AF647 fluorescence was visualized (after clearing). Panels show maximum-z projections of confocal image stacks prepared on a Leica SP8. SNR ± SD values are indicated on upper right angles of each panel. (B-C) EDL whole mounts were cleared using the X-CLARITY protocol and then stained with WGA-488. After overnight incubation in 88 % glycerol, WGA-488 fluorescence was measured. (A) Maximum-z projection of a representative confocal image stack prepared on a Leica SP8. Scale bar, 200 µm. (B) Detail from the boxed region in A. Arrowheads, NMJs. Scale bar, 50 µm.

**Supplementary Figure 2.** **SNR measurements for MYOCLEAR samples stained before or after the clearing process.** EDL muscles were cleared using the MYOCLEAR protocol. BGT-AF647 based NMJ staining either occurred before or after muscle optical clearing. The graph reports SNR values obtained for NMJ fluorescence signals from three different muscles per group, as a function of depth from the muscle surface. Triangles, diamonds, and circles indicate the different muscles per group. Black show values from samples that were first stained, red symbols depict values from samples that were first cleared. Each data point is the mean of at least three NMJ SNR values.

## Supplementary Videos

**Supplementary Video 1.** **EDL-whole mount analysis of myonuclei and NMJs is feasible using MYOCLEAR.** EDL whole mounts were cleared using the MYOCLEAR protocol and then stained for nuclei with Draq5 and for NMJs with BGT-AF647. After overnight incubation in 88 % glycerol, Draq5 and BGT-AF647 fluorescence signals were measured. The video is based on the data stack used in Fig. 6 and first shows a fly-through from top to bottom of the muscle and back. Then, the data stack is rotated by 90 ° to give a side view. Again, a whole fly-through is shown. Finally, the stack rotates back to the original position. Myonuclei and NMJs are depicted in red and green, respectively. On the left side, green autofluorescence of the thread holding the muscle in place during image acquisition is visible.

**Supplementary Video 2.** **Graphical 3D-representation of NMJs positions in EDL whole mount reveals the formation of a sinuous synapse band.** EDL whole mounts were cleared using the MYOCLEAR protocol and then stained for NMJs with BGT-AF647. After overnight incubation in 88 % glycerol, Draq5 and BGT-AF647 fluorescence signals were measured. The xyz-position of each NMJ was recorded using the multi-point tool in ImageJ (purple spots) or a preliminary automated detection algorithm (yellow spots) and then plotted into a 3D coordinate system. The video shows a rotation view of the detected synapse positions. The same data set as in Fig. 7A was used.
