# Supplementary figures and images for: A Novel Optical Tissue Clearing Protocol for Mouse Skeletal Muscle to Visualize Endplates in Their Tissue Context
Source: Front Cell Neurosci. 2019 Feb 27;13:49. doi: 10.3389/fncel.2019.00049 (PMC6401545; doi:10.3389/fncel.2019.00049)

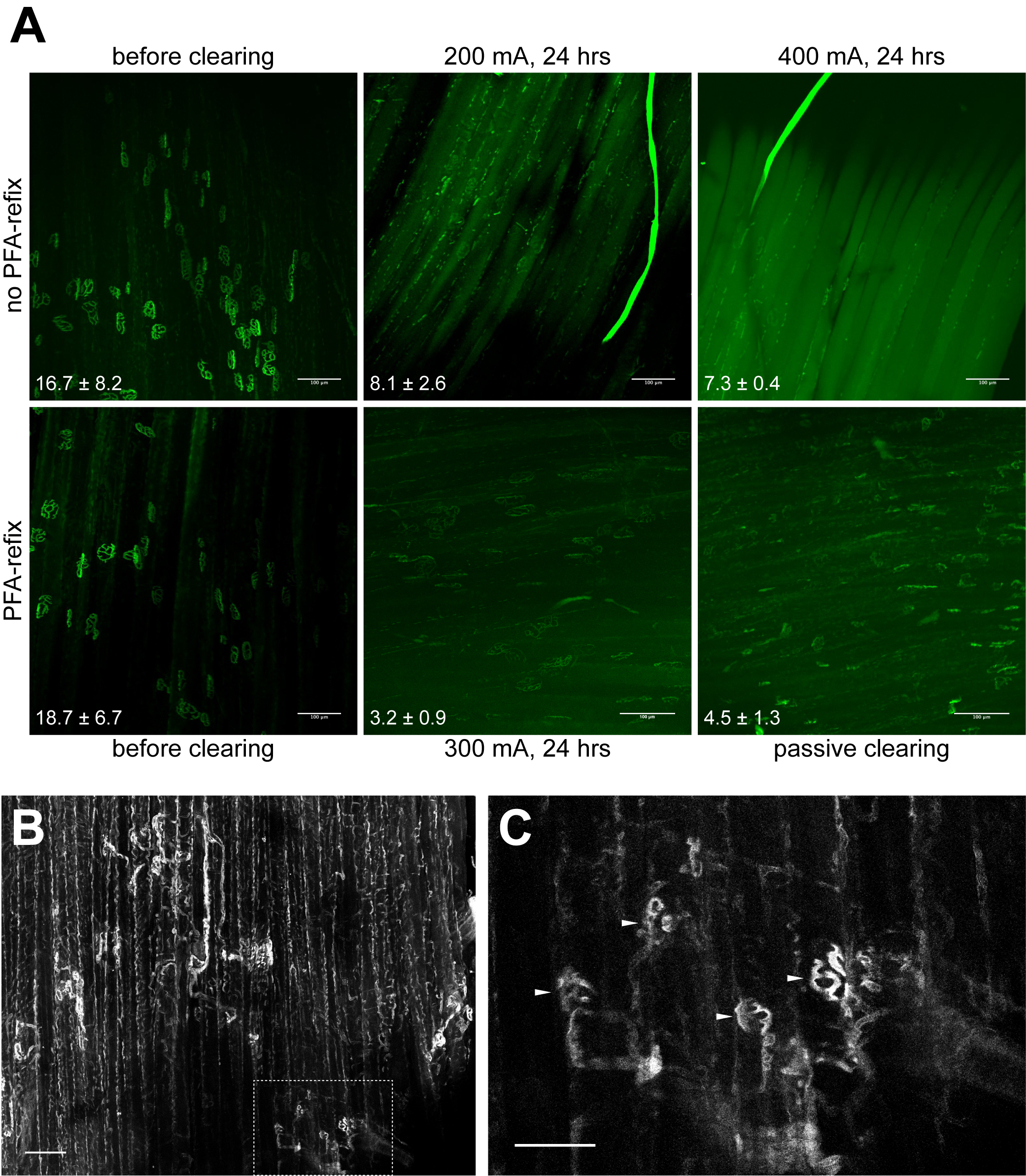

Supplement: Supplementary file 4 [file Image_1.JPEG]

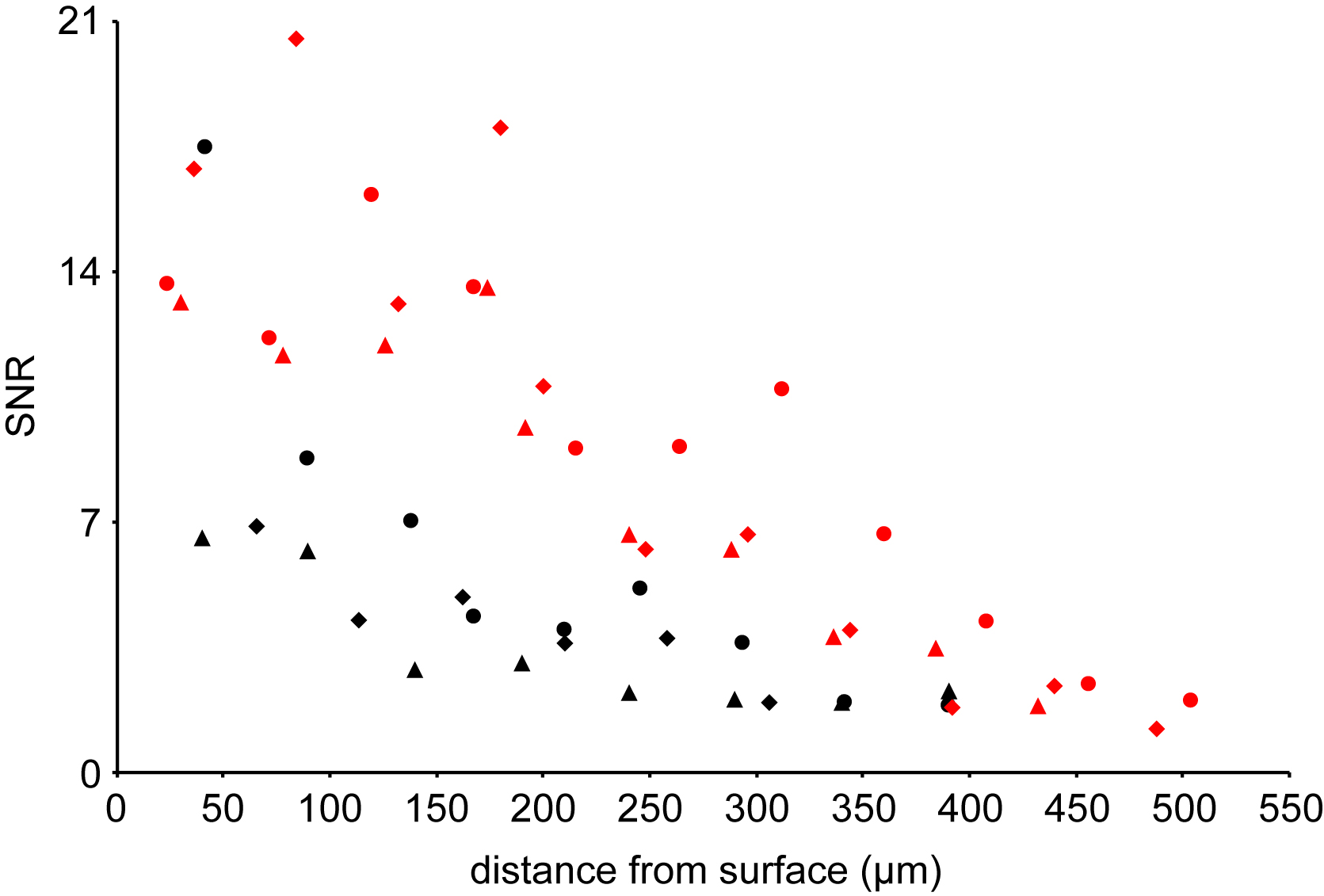

Supplement: Supplementary file 5 [file Image_2.JPEG]
